# 4-Methyl­benzoic acid–*N*′-[(*E*)-4-methyl­benzyl­idene]pyridine-4-carbohydrazide–water (1/1/1)

**DOI:** 10.1107/S1600536812016868

**Published:** 2012-04-21

**Authors:** Hoong-Kun Fun, Chin Wei Ooi, Divya N. Shetty, B. Narayana, B. K. Sarojini

**Affiliations:** a X-ray Crystallography Unit, School of Physics, Universiti Sains Malaysia, 11800 USM, Penang, Malaysia; b Department of Studies in Chemistry, Mangalore University, Mangalagangotri 574 199, India; c Department of Chemistry, P.A. College of Engineering, Nadupadavu, Mangalore 574 153, India

## Abstract

In the title hydrated 1:1 adduct, C_8_H_8_O_2_·C_14_H_13_N_3_O·H_2_O, the Schiff base mol­ecule exists in an *E* conformation with respect to the N=C bond [1.2843 (13) Å] and the dihedral angle between the pyridine ring and the benzene ring is 1.04 (5)°. In the crystal, mol­ecules are linked by N—H⋯O, C—H⋯O, O—H⋯O and O—H⋯N hydrogen bonds into sheets lying parallel to the *ab* plane. The crystal structure also features π–π inter­actions with centroid–centroid distances of 3.6224 (6) and 3.7121 (6) Å.

## Related literature

For related structures, see: Jing *et al.* (2005 ▶); Wang *et al.* (2007 ▶). For the stability of the temperature controller used in the data collection, see: Cosier & Glazer (1986 ▶). 
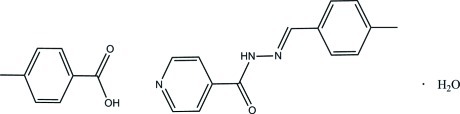


## Experimental

### 

#### Crystal data

C_8_H_8_O_2_·C_14_H_13_N_3_O·H_2_O
*M* *_r_* = 393.43
Orthorhombic, 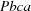

*a* = 7.3199 (4) Å
*b* = 11.6311 (6) Å
*c* = 45.875 (2) Å
*V* = 3905.7 (4) Å^3^
*Z* = 8
Mo *K*α radiation
μ = 0.09 mm^−1^
*T* = 100 K
0.31 × 0.22 × 0.13 mm

#### Data collection

Bruker APEX DUO CCD diffractometer
Absorption correction: multi-scan (*SADABS*; Bruker, 2009 ▶) *T*_min_ = 0.972, *T*_max_ = 0.988
59762 measured reflections
5751 independent reflections
5011 reflections with *I* > 2σ(*I*)
*R*_int_ = 0.035

#### Refinement

*R*[*F* ^2^ > 2σ(*F* ^2^)] = 0.040
*wR*(*F* ^2^) = 0.108
*S* = 1.07
5751 reflections
280 parameters
H atoms treated by a mixture of independent and constrained refinement
Δρ_max_ = 0.41 e Å^−3^
Δρ_min_ = −0.23 e Å^−3^

### 

Data collection: *APEX2* (Bruker, 2009 ▶); cell refinement: *SAINT* (Bruker, 2009 ▶); data reduction: *SAINT*; program(s) used to solve structure: *SHELXTL* (Sheldrick, 2008 ▶); program(s) used to refine structure: *SHELXTL*; molecular graphics: *SHELXTL*; software used to prepare material for publication: *SHELXTL* and *PLATON* (Spek, 2009 ▶).

## Supplementary Material

Crystal structure: contains datablock(s) global, I. DOI: 10.1107/S1600536812016868/hb6743sup1.cif

Structure factors: contains datablock(s) I. DOI: 10.1107/S1600536812016868/hb6743Isup2.hkl

Supplementary material file. DOI: 10.1107/S1600536812016868/hb6743Isup3.cml

Additional supplementary materials:  crystallographic information; 3D view; checkCIF report

## Figures and Tables

**Table 1:** Hydrogen-bond geometry (Å, °)

*D*—H⋯*A*	*D*—H	H⋯*A*	*D*⋯*A*	*D*—H⋯*A*
O3—H1*O*3⋯N1	0.99 (2)	1.66 (2)	2.6347 (13)	168 (2)
O1*W*—H1*W*1⋯O1	0.887 (17)	1.927 (17)	2.7974 (11)	166.7 (16)
O1*W*—H2*W*1⋯N3^i^	0.89 (2)	2.14 (2)	3.0231 (12)	168.7 (17)
N2—H1*N*2⋯O1*W*^ii^	0.873 (17)	1.988 (17)	2.8120 (12)	157.0 (14)
C4—H4*A*⋯O1^iii^	0.95	2.40	3.2796 (13)	154
C10—H10*A*⋯O2^iv^	0.95	2.51	3.4188 (13)	160

Symmetry codes: (i) 
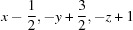
; (ii) 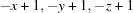; (iii) 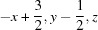; (iv) 
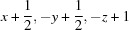
.

**Fig. 1.**
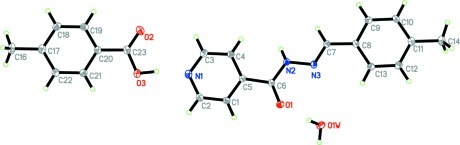
The molecular structure of the title compound, showing 50% probability displacement ellipsoids.

**Fig. 2.**
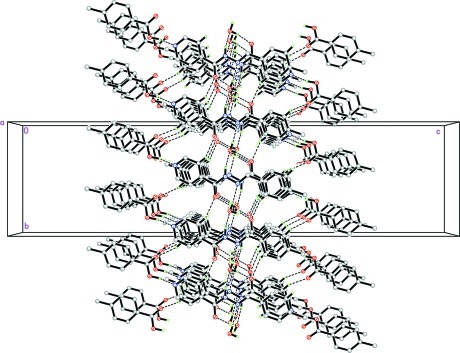
The crystal packing of the title compound, viewed along the a axis. H atoms not involved in the intermolecular interactions (dashed lines) have been omitted for clarity.

## supplementary crystallographic information

### Comment

The structures of isoniazid and its various derivatives have been described previously (Wang *et al.*, 2007; Jing *et al.*, 2005). Here, the synthesis and crystal structure of its Schiff base derivative (I) is reported. The Schiff base, *N*'-[(*E*)-(4-methylphenyl)methylidene]pyridine-4- carbohydrazide was synthesized by the condensation of isoniazid with 4-methylbenzaldehyde in absolute alcohol in presence of hydrochloric acid. During the crystallization, the synthesized Schiff base crystallized with 4-methyl benzoic acid (which is a side product obtained by the auto-oxidation of unreacted 4-methyl benzaldehyde) and one molecule of water to form title compound (I) (Fig. 1).

The Schiff base molecule exists in an *E* conformation with respect to the N3 ═ C7 bond [N3 ═C7 = 1.2843 (13) Å; torsion angle N2—N3—C7—C8 = -178.00 (8)°]. The pyridine ring (N1/C1–C5) is essentially planar with a maximum deviation of 0.002 (1) Å at atoms C1 and C5. There is a slight inclination between the pyridine ring and the benzene ring (C8—C13), as indicated by the dihedral angle formed of 1.04 (5) °. The bond lengths and angles are comparable with those in related structures (Wang *et al.*, 2007 and Jing *et al.*, 2005).

In the crystal (Fig. 2), the molecules are linked *via* N2—H1N2···O1W, C4—H4A···O1, C10—H10A···O2, O1W—H1W1···O1, O1W—H2W1···N3 and O3—H1O3···N1 hydrogen bonds (Table 1) into two-dimensional networks parallel to the *ab* plane. The crystal structure also features π—π interactions with *Cg*1···*Cg*2 = 3.6224 (6) Å [symmetry code: 1-*X*, 1-Y, 1-*Z*] and *Cg*3···*Cg*3 = 3.7121 (6) Å [symmetry code: -1/2+*X*, Y, 1/2-*Z*], where *Cg*1, *Cg*2 and *Cg*3 are the centroids of N1/C1–C5, C8–C13 and C17–C22 rings, respectively.

### Experimental

A mixture of isoniazid (1.4 g, 0.01 mol) and 4-methylbenzaldehyde (1.2 g, 0.01 mol) in 15 ml of absolute alcohol containing two drops of hydrochloric acid was refluxed for about 3 h. On cooling, a solid was separated out. The solid was filtered out and recrystallized from DMF. Colourless blocks of (I) were grown from DMF by slow evaporation method. During the crystallization, the synthesized Schiff base was crystallized with 4-methyl benzoic acid (which was a side product obtained by the auto-oxidation of unreacted 4-methyl benzaldehyde) and one molecule of water to form the title compound (I). (*M.p*: 428 K).

### Refinement

The O– and N– bound H atoms were located in a difference Fourier map and refine freely [O—H = 0.884 (18) - 0.99 (2) Å and N—H 0.875 (17) Å]. The remaining H atoms were positioned geometrically and refined using a riding model with *U*_iso_(H) = 1.2 or 1.5*U*_eq_(C) (C—H = 0.95 and 0.98 Å). A rotating group model was applied to the methyl groups. In the final refinement, one outlier (4 1 22) was omitted.

### Crystal data

**Table:**

C_8_H_8_O_2_·C_14_H_13_N_3_O·H_2_O	*F*(000) = 1664
*M**_r_* = 393.43	*D*_x_ = 1.338 Mg m^−^^3^
Orthorhombic, *P**b**c**a*	Mo *K*α radiation, λ = 0.71073 Å
Hall symbol: -P 2ac 2ab	Cell parameters from 9870 reflections
*a* = 7.3199 (4) Å	θ = 2.7–30.1°
*b* = 11.6311 (6) Å	µ = 0.09 mm^−^^1^
*c* = 45.875 (2) Å	*T* = 100 K
*V* = 3905.7 (4) Å^3^	Block, colourless
*Z* = 8	0.31 × 0.22 × 0.13 mm

### Data collection

**Table:**

Bruker APEX DUO CCD diffractometer	5751 independent reflections
Radiation source: fine-focus sealed tube	5011 reflections with *I* > 2σ(*I*)
Graphite monochromator	*R*_int_ = 0.035
φ and ω scans	θ_max_ = 30.1°, θ_min_ = 1.8°
Absorption correction: multi-scan (*SADABS*; Bruker, 2009)	*h* = −10→10
*T*_min_ = 0.972, *T*_max_ = 0.988	*k* = −16→15
59762 measured reflections	*l* = −61→64

### Refinement

**Table:**

Refinement on *F*^2^	Primary atom site location: structure-invariant direct methods
Least-squares matrix: full	Secondary atom site location: difference Fourier map
*R*[*F*^2^ > 2σ(*F*^2^)] = 0.040	Hydrogen site location: inferred from neighbouring sites
*wR*(*F*^2^) = 0.108	H atoms treated by a mixture of independent and constrained refinement
*S* = 1.07	*w* = 1/[σ^2^(*F*_o_^2^) + (0.051*P*)^2^ + 1.7498*P*] where *P* = (*F*_o_^2^ + 2*F*_c_^2^)/3
5751 reflections	(Δ/σ)_max_ = 0.001
280 parameters	Δρ_max_ = 0.41 e Å^−^^3^
0 restraints	Δρ_min_ = −0.23 e Å^−^^3^

### Special details

**Table:**

Experimental. The crystal was placed in the cold stream of an Oxford Cryosystems Cobra open-flow nitrogen cryostat (Cosier & Glazer, 1986) operating at 100.0 (1) K.
Geometry. All e.s.d.'s (except the e.s.d. in the dihedral angle between two l.s. planes) are estimated using the full covariance matrix. The cell e.s.d.'s are taken into account individually in the estimation of e.s.d.'s in distances, angles and torsion angles; correlations between e.s.d.'s in cell parameters are only used when they are defined by crystal symmetry. An approximate (isotropic) treatment of cell e.s.d.'s is used for estimating e.s.d.'s involving l.s. planes.
Refinement. Refinement of *F*^2^ against ALL reflections. The weighted *R*-factor *wR* and goodness of fit *S* are based on *F*^2^, conventional *R*-factors *R* are based on *F*, with *F* set to zero for negative *F*^2^. The threshold expression of *F*^2^ > σ(*F*^2^) is used only for calculating *R*-factors(gt) *etc*. and is not relevant to the choice of reflections for refinement. *R*-factors based on *F*^2^ are statistically about twice as large as those based on *F*, and *R*- factors based on ALL data will be even larger.

### Fractional atomic coordinates and isotropic or equivalent isotropic displacement parameters (Å^2^)

**Table:**

	*x*	*y*	*z*	*U*_iso_*/*U*_eq_	
O1W	0.43207 (11)	0.73964 (7)	0.507362 (18)	0.01826 (16)	
O1	0.63620 (11)	0.64905 (6)	0.461214 (16)	0.01601 (15)	
O2	0.69422 (12)	0.14784 (7)	0.336901 (17)	0.02270 (18)	
O3	0.56737 (13)	0.31295 (8)	0.322091 (18)	0.0261 (2)	
N1	0.59728 (12)	0.38603 (8)	0.376242 (19)	0.01606 (17)	
N2	0.71637 (12)	0.47981 (7)	0.482605 (18)	0.01298 (16)	
N3	0.75142 (12)	0.53545 (7)	0.508766 (18)	0.01307 (16)	
C1	0.56364 (13)	0.54599 (9)	0.40841 (2)	0.01386 (18)	
H1A	0.5261	0.6233	0.4113	0.017*	
C2	0.54681 (14)	0.49497 (9)	0.38132 (2)	0.0164 (2)	
H2A	0.4976	0.5388	0.3657	0.020*	
C3	0.66639 (13)	0.32491 (9)	0.39840 (2)	0.01493 (19)	
H3A	0.7018	0.2476	0.3949	0.018*	
C4	0.68877 (13)	0.36905 (9)	0.42629 (2)	0.01328 (18)	
H4A	0.7385	0.3232	0.4415	0.016*	
C5	0.63617 (12)	0.48261 (8)	0.43136 (2)	0.01171 (17)	
C6	0.66039 (13)	0.54445 (8)	0.45976 (2)	0.01197 (18)	
C7	0.80639 (13)	0.46935 (9)	0.52941 (2)	0.01297 (18)	
H7A	0.8232	0.3898	0.5256	0.016*	
C8	0.84364 (13)	0.51370 (8)	0.55861 (2)	0.01218 (18)	
C9	0.92034 (13)	0.43970 (9)	0.57926 (2)	0.01375 (18)	
H9A	0.9517	0.3634	0.5738	0.017*	
C10	0.95145 (13)	0.47632 (9)	0.60776 (2)	0.01496 (19)	
H10A	1.0041	0.4249	0.6215	0.018*	
C11	0.90594 (13)	0.58772 (9)	0.61626 (2)	0.01477 (19)	
C12	0.82840 (13)	0.66163 (9)	0.59548 (2)	0.01501 (19)	
H12A	0.7962	0.7377	0.6010	0.018*	
C13	0.79782 (13)	0.62599 (9)	0.56710 (2)	0.01372 (18)	
H13A	0.7458	0.6776	0.5534	0.016*	
C14	0.93757 (16)	0.62885 (10)	0.64699 (2)	0.0211 (2)	
H14A	1.0008	0.5689	0.6581	0.032*	
H14B	0.8199	0.6458	0.6562	0.032*	
H14C	1.0126	0.6986	0.6466	0.032*	
C16	0.65514 (17)	0.08019 (11)	0.19543 (2)	0.0240 (2)	
H16A	0.6553	−0.0038	0.1936	0.036*	
H16B	0.7672	0.1113	0.1868	0.036*	
H16C	0.5488	0.1117	0.1852	0.036*	
C17	0.64625 (14)	0.11288 (9)	0.22720 (2)	0.0168 (2)	
C18	0.69565 (14)	0.03460 (9)	0.24886 (3)	0.0182 (2)	
H18A	0.7340	−0.0405	0.2435	0.022*	
C19	0.68955 (14)	0.06485 (9)	0.27810 (2)	0.0166 (2)	
H19A	0.7241	0.0107	0.2926	0.020*	
C20	0.63260 (13)	0.17483 (9)	0.28622 (2)	0.01416 (19)	
C21	0.57996 (14)	0.25295 (9)	0.26468 (2)	0.01573 (19)	
H21A	0.5387	0.3275	0.2700	0.019*	
C22	0.58768 (14)	0.22215 (9)	0.23551 (2)	0.0169 (2)	
H22A	0.5526	0.2761	0.2210	0.020*	
C23	0.63459 (14)	0.20873 (9)	0.31746 (2)	0.01617 (19)	
H1N2	0.698 (2)	0.4055 (15)	0.4832 (4)	0.031 (4)*	
H2W1	0.366 (3)	0.8001 (16)	0.5016 (4)	0.042 (5)*	
H1W1	0.504 (2)	0.7217 (15)	0.4926 (4)	0.034 (4)*	
H1O3	0.580 (3)	0.3298 (19)	0.3432 (5)	0.063 (6)*	

### Atomic displacement parameters (Å^2^)

**Table:**

	*U*^11^	*U*^22^	*U*^33^	*U*^12^	*U*^13^	*U*^23^
O1W	0.0218 (4)	0.0135 (4)	0.0194 (4)	0.0021 (3)	0.0042 (3)	0.0000 (3)
O1	0.0220 (4)	0.0109 (3)	0.0151 (3)	0.0003 (3)	−0.0003 (3)	0.0003 (3)
O2	0.0291 (4)	0.0237 (4)	0.0153 (4)	0.0021 (3)	−0.0009 (3)	0.0034 (3)
O3	0.0398 (5)	0.0242 (4)	0.0144 (4)	0.0109 (4)	−0.0047 (3)	−0.0053 (3)
N1	0.0162 (4)	0.0189 (4)	0.0131 (4)	−0.0010 (3)	0.0009 (3)	−0.0011 (3)
N2	0.0169 (4)	0.0108 (4)	0.0112 (4)	−0.0005 (3)	−0.0015 (3)	−0.0002 (3)
N3	0.0144 (4)	0.0138 (4)	0.0110 (4)	−0.0012 (3)	−0.0006 (3)	−0.0006 (3)
C1	0.0139 (4)	0.0137 (4)	0.0139 (4)	0.0008 (3)	0.0002 (3)	0.0016 (3)
C2	0.0175 (5)	0.0191 (5)	0.0126 (4)	0.0003 (4)	−0.0011 (3)	0.0022 (4)
C3	0.0148 (4)	0.0146 (4)	0.0154 (5)	−0.0005 (3)	0.0017 (3)	−0.0017 (4)
C4	0.0131 (4)	0.0131 (4)	0.0136 (4)	0.0003 (3)	0.0005 (3)	0.0008 (3)
C5	0.0105 (4)	0.0130 (4)	0.0116 (4)	−0.0011 (3)	0.0008 (3)	0.0004 (3)
C6	0.0110 (4)	0.0132 (4)	0.0117 (4)	−0.0010 (3)	0.0008 (3)	0.0005 (3)
C7	0.0132 (4)	0.0124 (4)	0.0133 (4)	−0.0001 (3)	0.0003 (3)	−0.0002 (3)
C8	0.0111 (4)	0.0140 (4)	0.0115 (4)	−0.0011 (3)	0.0000 (3)	0.0009 (3)
C9	0.0134 (4)	0.0134 (4)	0.0145 (4)	0.0003 (3)	0.0000 (3)	0.0011 (3)
C10	0.0138 (4)	0.0182 (5)	0.0129 (4)	0.0000 (3)	−0.0012 (3)	0.0029 (4)
C11	0.0127 (4)	0.0187 (5)	0.0128 (4)	−0.0029 (3)	0.0011 (3)	0.0001 (4)
C12	0.0153 (4)	0.0136 (4)	0.0161 (5)	−0.0016 (3)	0.0016 (3)	−0.0011 (4)
C13	0.0137 (4)	0.0133 (4)	0.0141 (4)	−0.0004 (3)	0.0002 (3)	0.0014 (3)
C14	0.0232 (5)	0.0263 (6)	0.0137 (5)	−0.0023 (4)	0.0000 (4)	−0.0028 (4)
C16	0.0248 (5)	0.0304 (6)	0.0167 (5)	−0.0025 (4)	0.0010 (4)	−0.0073 (4)
C17	0.0131 (4)	0.0206 (5)	0.0166 (5)	−0.0027 (4)	0.0001 (3)	−0.0043 (4)
C18	0.0155 (4)	0.0171 (5)	0.0221 (5)	0.0008 (4)	−0.0011 (4)	−0.0051 (4)
C19	0.0147 (4)	0.0157 (5)	0.0193 (5)	0.0002 (3)	−0.0018 (4)	0.0003 (4)
C20	0.0123 (4)	0.0163 (5)	0.0139 (4)	−0.0014 (3)	−0.0001 (3)	−0.0010 (3)
C21	0.0158 (4)	0.0150 (4)	0.0165 (5)	0.0006 (3)	−0.0003 (3)	−0.0010 (4)
C22	0.0172 (4)	0.0188 (5)	0.0147 (4)	−0.0009 (4)	−0.0015 (4)	0.0001 (4)
C23	0.0149 (4)	0.0189 (5)	0.0147 (5)	−0.0017 (4)	0.0002 (3)	0.0000 (4)

### Geometric parameters (Å, º)

**Table:**

O1W—H2W1	0.89 (2)	C9—H9A	0.9500
O1W—H1W1	0.884 (18)	C10—C11	1.3935 (15)
O1—C6	1.2312 (12)	C10—H10A	0.9500
O2—C23	1.2197 (13)	C11—C12	1.4035 (14)
O3—C23	1.3254 (13)	C11—C14	1.5068 (14)
O3—H1O3	0.99 (2)	C12—C13	1.3845 (14)
N1—C3	1.3396 (13)	C12—H12A	0.9500
N1—C2	1.3402 (14)	C13—H13A	0.9500
N2—C6	1.3533 (12)	C14—H14A	0.9800
N2—N3	1.3874 (11)	C14—H14B	0.9800
N2—H1N2	0.875 (17)	C14—H14C	0.9800
N3—C7	1.2843 (13)	C16—C17	1.5073 (15)
C1—C2	1.3825 (14)	C16—H16A	0.9800
C1—C5	1.3908 (13)	C16—H16B	0.9800
C1—H1A	0.9500	C16—H16C	0.9800
C2—H2A	0.9500	C17—C22	1.3944 (15)
C3—C4	1.3884 (14)	C17—C18	1.3956 (16)
C3—H3A	0.9500	C18—C19	1.3874 (15)
C4—C5	1.3954 (13)	C18—H18A	0.9500
C4—H4A	0.9500	C19—C20	1.3960 (14)
C5—C6	1.4985 (13)	C19—H19A	0.9500
C7—C8	1.4612 (13)	C20—C21	1.3966 (14)
C7—H7A	0.9500	C20—C23	1.4863 (14)
C8—C9	1.3978 (13)	C21—C22	1.3867 (14)
C8—C13	1.4035 (14)	C21—H21A	0.9500
C9—C10	1.3936 (14)	C22—H22A	0.9500
			
H2W1—O1W—H1W1	106.4 (16)	C13—C12—C11	121.37 (9)
C23—O3—H1O3	107.7 (13)	C13—C12—H12A	119.3
C3—N1—C2	118.28 (9)	C11—C12—H12A	119.3
C6—N2—N3	117.83 (8)	C12—C13—C8	120.06 (9)
C6—N2—H1N2	121.6 (11)	C12—C13—H13A	120.0
N3—N2—H1N2	117.7 (11)	C8—C13—H13A	120.0
C7—N3—N2	114.61 (8)	C11—C14—H14A	109.5
C2—C1—C5	119.15 (9)	C11—C14—H14B	109.5
C2—C1—H1A	120.4	H14A—C14—H14B	109.5
C5—C1—H1A	120.4	C11—C14—H14C	109.5
N1—C2—C1	122.52 (9)	H14A—C14—H14C	109.5
N1—C2—H2A	118.7	H14B—C14—H14C	109.5
C1—C2—H2A	118.7	C17—C16—H16A	109.5
N1—C3—C4	123.20 (9)	C17—C16—H16B	109.5
N1—C3—H3A	118.4	H16A—C16—H16B	109.5
C4—C3—H3A	118.4	C17—C16—H16C	109.5
C3—C4—C5	118.11 (9)	H16A—C16—H16C	109.5
C3—C4—H4A	120.9	H16B—C16—H16C	109.5
C5—C4—H4A	120.9	C22—C17—C18	118.66 (10)
C1—C5—C4	118.74 (9)	C22—C17—C16	120.50 (10)
C1—C5—C6	116.68 (9)	C18—C17—C16	120.84 (10)
C4—C5—C6	124.52 (9)	C19—C18—C17	120.98 (10)
O1—C6—N2	123.40 (9)	C19—C18—H18A	119.5
O1—C6—C5	120.29 (9)	C17—C18—H18A	119.5
N2—C6—C5	116.26 (9)	C18—C19—C20	120.00 (10)
N3—C7—C8	121.54 (9)	C18—C19—H19A	120.0
N3—C7—H7A	119.2	C20—C19—H19A	120.0
C8—C7—H7A	119.2	C19—C20—C21	119.33 (9)
C9—C8—C13	118.74 (9)	C19—C20—C23	119.83 (9)
C9—C8—C7	118.62 (9)	C21—C20—C23	120.81 (9)
C13—C8—C7	122.57 (9)	C22—C21—C20	120.23 (10)
C10—C9—C8	120.88 (9)	C22—C21—H21A	119.9
C10—C9—H9A	119.6	C20—C21—H21A	119.9
C8—C9—H9A	119.6	C21—C22—C17	120.79 (10)
C11—C10—C9	120.50 (9)	C21—C22—H22A	119.6
C11—C10—H10A	119.8	C17—C22—H22A	119.6
C9—C10—H10A	119.8	O2—C23—O3	123.14 (10)
C10—C11—C12	118.44 (9)	O2—C23—C20	123.68 (10)
C10—C11—C14	121.35 (9)	O3—C23—C20	113.18 (9)
C12—C11—C14	120.21 (10)		
			
C6—N2—N3—C7	−179.09 (9)	C9—C10—C11—C12	0.00 (15)
C3—N1—C2—C1	0.06 (15)	C9—C10—C11—C14	179.69 (10)
C5—C1—C2—N1	0.23 (15)	C10—C11—C12—C13	−0.28 (15)
C2—N1—C3—C4	−0.23 (15)	C14—C11—C12—C13	−179.97 (9)
N1—C3—C4—C5	0.11 (15)	C11—C12—C13—C8	0.34 (15)
C2—C1—C5—C4	−0.34 (14)	C9—C8—C13—C12	−0.12 (14)
C2—C1—C5—C6	176.80 (9)	C7—C8—C13—C12	176.90 (9)
C3—C4—C5—C1	0.18 (14)	C22—C17—C18—C19	−0.95 (15)
C3—C4—C5—C6	−176.72 (9)	C16—C17—C18—C19	179.28 (10)
N3—N2—C6—O1	−1.32 (14)	C17—C18—C19—C20	0.28 (16)
N3—N2—C6—C5	175.99 (8)	C18—C19—C20—C21	0.81 (15)
C1—C5—C6—O1	−8.01 (13)	C18—C19—C20—C23	−177.12 (9)
C4—C5—C6—O1	168.95 (9)	C19—C20—C21—C22	−1.24 (15)
C1—C5—C6—N2	174.60 (9)	C23—C20—C21—C22	176.67 (9)
C4—C5—C6—N2	−8.45 (14)	C20—C21—C22—C17	0.57 (15)
N2—N3—C7—C8	−178.00 (8)	C18—C17—C22—C21	0.52 (15)
N3—C7—C8—C9	−173.70 (9)	C16—C17—C22—C21	−179.71 (10)
N3—C7—C8—C13	9.28 (15)	C19—C20—C23—O2	4.58 (16)
C13—C8—C9—C10	−0.15 (14)	C21—C20—C23—O2	−173.33 (10)
C7—C8—C9—C10	−177.30 (9)	C19—C20—C23—O3	−176.40 (9)
C8—C9—C10—C11	0.22 (15)	C21—C20—C23—O3	5.70 (14)

### Hydrogen-bond geometry (Å, º)

**Table:**

*D*—H···*A*	*D*—H	H···*A*	*D*···*A*	*D*—H···*A*
O3—H1*O*3···N1	0.99 (2)	1.66 (2)	2.6347 (13)	168 (2)
O1*W*—H1*W*1···O1	0.887 (17)	1.927 (17)	2.7974 (11)	166.7 (16)
O1*W*—H2*W*1···N3^i^	0.89 (2)	2.14 (2)	3.0231 (12)	168.7 (17)
N2—H1*N*2···O1*W*^ii^	0.873 (17)	1.988 (17)	2.8120 (12)	157.0 (14)
C4—H4*A*···O1^iii^	0.95	2.40	3.2796 (13)	154
C10—H10*A*···O2^iv^	0.95	2.51	3.4188 (13)	160

Symmetry codes: (i) *x*−1/2, −*y*+3/2, −*z*+1; (ii) −*x*+1, −*y*+1, −*z*+1; (iii) −*x*+3/2, *y*−1/2, *z*; (iv) *x*+1/2, −*y*+1/2, −*z*+1.
